# Biological Synthesis of CdS/CdSe Core/Shell Nanoparticles and Its Application in Quantum Dot Sensitized Solar Cells

**DOI:** 10.3389/fmicb.2019.01587

**Published:** 2019-07-11

**Authors:** Nicolás Órdenes-Aenishanslins, Giovanna Anziani-Ostuni, Carolina P. Quezada, Rodrigo Espinoza-González, Denisse Bravo, José M. Pérez-Donoso

**Affiliations:** ^1^BioNanotechnology and Microbiology Laboratory, Center for Bioinformatics and Integrative Biology, Facultad de Ciencias de la Vida, Universidad Andrés Bello, Santiago, Chile; ^2^Departamento de Ingeniería Química, Biotecnología y Materiales, Facultad de Ciencias Físicas y Matemáticas, Universidad de Chile, Santiago, Chile; ^3^Laboratorio de Microbiología Oral, Facultad de Odontología, Universidad de Chile, Santiago, Chile

**Keywords:** nanoparticle biosynthesis, fluorescent nanoparticles, QDSSC, green photovoltaic devices, core shell quantum dots

## Abstract

In the present work, we report the use of bacterial cells for the production of CdS/CdSe Core/Shell quantum dots (QDs), a complex nanostructure specially designed to improve their performance as photosensitizer in photovoltaic devices. The method requires the incorporation of L-cysteine, CdCl_2_ and Na_2_SeO_3_ to *Escherichia coli* cultures and allows a tight control of QDs properties. The obtained CdS/CdSe QDs were photophysically and structurally characterized. When compared to CdS QDs, the classical shift in the UV-visible spectra of Core/Shell nanostructures was observed in CdS/CdSe QDs. The nanosize, structure, and composition of Core/Shell QDs were confirmed by TEM and EDS analysis. QDs presented a size of approximately 12 nm (CdS) and 17 nm (CdS/CdSe) as determined by dynamic light scattering (DLS), whereas the fourier transform infrared (FTIR) spectra allowed to distinguish the presence of different biomolecules bound to both types of nanoparticles. An increased photostability was observed in CdS/CdSe nanoparticles when compared to CdS QDs. Finally, biosynthesized CdS/CdSe Core/Shell QDs were used as photosensitizers for quantum dots sensitized solar cells (QDSSCs) and their photovoltaic parameters determined. As expected, the efficiency of solar cells sensitized with biological CdS/CdSe QDs increased almost 2.5 times when compared to cells sensitized with CdS QDs. This work is the first report of biological synthesis of CdS/CdSe Core/Shell QDs using bacterial cells and represents a significant contribution to the development of green and low-cost photovoltaic technologies.

## Introduction

During the last decade, the interest in replacing fossil fuels with non-conventional renewable energies (NCRE) has grown worldwide (Hoekstra and Wiedmann, 2014). Among all NCRE, sunlight is particularly relevant because is the more abundant, clean, and available source of energy in our planet (Schiermeier et al., 2008). Based on this, photovoltaic market has been constantly growing and evolving to solve the requirements for sustainable and clean energy generation. However, current photovoltaic technologies have some limitations like high production costs, large quantities of materials required, and the emission of toxic compounds associated with its manufacture (Parida et al., 2011).

First generation solar cells, built mainly of silicon, reach near 25% of efficiency and dominate the market, but they involve high production costs, and environmental impact (Green et al., 2015). The second generation of solar cells, based on thin layers of semi-conductor materials (mainly metal alloys made of Cu, In, Ga, and As), in general present similar efficiencies and slightly lower production costs, however, this technology still have a high environmental impact (Green et al., 2015). As a response to these requirements the third generation of solar cells emerged, especially the quantum dot sensitized solar cells (QDSSCs) (O’Regan and Grätzel, 1991; Grätzel, 2003; Rühle et al., 2010; Jun et al., 2013; Pan et al., 2018). This type of solar cell displays the lowest rates of sulfur oxide, nitrogen oxide and carbon dioxide emission compared to other photovoltaic technologies (Tyagi et al., 2013). QDSSCs uses fluorescent semiconductor nanoparticles or quantum dots (QDs) that, due to their optoelectronic properties, are able to absorb light, and transfer electrons in their excited state to a TiO_2_ semiconductor. In addition, QDs exhibit high structural stability and a broad light absorption spectrum. Depending on the QD composition and nanostructure of the TiO_2_ layer, the efficiency range goes between 0.003 and <10% (Órdenes-Aenishanslins et al., 2014; Zhao et al., 2016).

**FIGURE 1:**
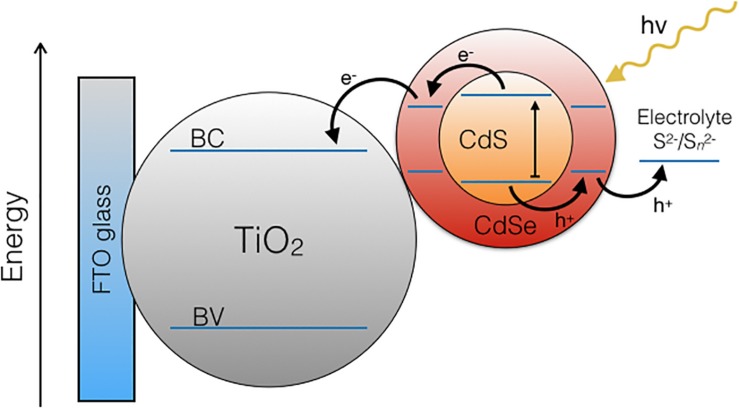
Schematic representation of the alignment of conduction bands in an electrode made of TiO_2_-CdS/CdSe in a QDSSC.

The QDs that presented the best performance in this type of cells are Core/Shell QDs, formed by a core and a shell of different composition and properties, including CdS/CdSe, CdTe/CdS, ZnSe/CdS, and PbS/CdS QDs, among others (Boyer et al., 2003; Lee and Lo, 2009; Jun et al., 2013; Lai et al., 2014; Qiu et al., 2017). The shell act as a layer that order and trap the electrons in the higher energetic states, helping the movement of charges to the electrode, and avoiding alternative decays (Reiss et al., 2009; Vasudevan et al., 2015). In the case of CdS/CdSe QDs coupled to a TiO_2_ semiconductor layer, the energy of their conduction bands forms a stepwise cascade, allowing an alignment that favors the correct transference of electrons on the electrode (Figure 1; Lee and Lo, 2009; Hossain et al., 2011).

This cascade is only possible with the configuration of CdS as the QD nucleus (core) and CdSe as the shell. Nevertheless, the configuration CdSe/CdS has been proved to have a high performance in QDSSCs when coated with a ZnS layer (Yu et al., 2012) and also can improve the absorption range of silicon cells (Lopez-Delgado et al., 2017).

In general, the synthesis of QDs involves complex chemical procedures, such as high temperatures, anaerobic and reactive conditions, and the generation of toxic residues. Consequently, in order to develop simpler an economical protocols for production with lower environmental impact, the use of microorganisms to biosynthesize these nanoparticles has gained attention (Li et al., 2011; Hulkoti and Taranath, 2014). In addition, it has been reported that biosynthesized QDs display unique properties such as increased acid and salt stability (Ulloa et al., 2016, 2018; Bruna et al., 2019). Until now, the microorganism most used for QDs biosynthesis is *Escherichia coli*, capable of producing CdS, CdSe, and CdTe QDs (Sweeney et al., 2004; Park et al., 2010; Monras et al., 2012; Yan et al., 2014). The capacity of this bacteria to biosynthesize QDs has been related to the presence of thiols, peptides and reductases present in the cell that allow the chelation of the metal, and the formation of core structures for the synthesis of nanoparticles (Bai et al., 2009; Mi et al., 2011; Raouf Hosseini and Nasiri Sarvi, 2015; Yan et al., 2017). Nowadays, the *E. coli* production of extracellular QDs is based on favoring the generation of the volatile thiol H_2_S from a sulfur source like glutathione or L-cysteine, and a metal salt (Gallardo et al., 2014; Plaza et al., 2016; Venegas et al., 2017). Despite all these advances, bacterial synthesis of more complex semiconductor nanostructures such as Core/Shell QDs has not been reported to date. Biological synthesis of QDs is a more difficult process than the biosynthesis of metal nanoparticles (e.g., Ag°, Se°, Cu°, and Au°) since the generation of hetero-nanostructures such as CdSe, CdTe, CdS, and Ag_2_S, among others, involves the interaction of elements in a certain oxidation state (e.g., Ag^+^, Cd^2+^, and Se^2–^). In this context, the use of microorganisms for the controlled generation of core shell hetero-nanostructures constitute an unexplored challenge (Gallardo et al., 2014; Plaza et al., 2016).

Based on this, the aim of this work was to develop a biological method to synthesize CdS/CdSe Core/Shell QDs and test their potential application in QDSSCs. We developed a CdS/CdSe QDs biosynthesis method based on the use of L-cysteine, CdCl_2_, and Na_2_SeO_3_. The influence of different buffers on the fluorescence of the QDs produced was evaluated, aiming to determine the conditions that favor their photostability. Biosynthesized Core/Shell QDs are functional in QDSSCs and their photovoltaic parameters showed an improvement when compared to solar cells sensitized with CdS QDs.

## Materials and Methods

### Biosynthesis of CdS and CdS/CdSe QDs

*Escherichia coli* BW25113 was grown in LB media at 37°C until stationary phase (OD_600_ 1.0). Cells were washed and resuspended in water or buffer (Borax-citrate pH 9.35; Tris–HCl pH 8.00 or Tris–citrate pH 8.00) and then exposed to 100 μM CdCl_2_ and different sulfur sources (Na_2_S_2_O_3_⋅5H_2_O, Na_2_SO_3_, Na_2_SO_4_, Na_2_S, L-methionine, or L-cysteine) at a final concentration of 1, 3, or 6 mM. Incubation times were 3, 6, and 24 h at 37°C. Then, cells were centrifuged 5 min at 27670 × *g* and supernatants exposed to UV light (MaestroGen UltraBright UV MLB-21, λ_exc_ = 365 nm). To evaluate the production of a CdSe shell on the CdS core, different concentrations of Na_2_SeO_3_ (1, 10, or 100 μM) were tested. After 120 min of incubation, cells were centrifuged 5 min at 27670 × *g* and supernatants collected for further analysis. For the experiments using dead cells, a stationary phase culture was incubated 1 h at 70°C.

### Purification and UV-Vis Characterization of CdS and CdS/CdSe QDs

Cells biosynthesizing QDs were centrifuged 5 min at 27670 × *g* and the QDs produced in the supernatant were purified by using 0.22 μm filters (Jet Bio- Filtration Co., Ltd.). Then, QDs were concentrated 20 times by centrifuging 40 min at 4000 × *g* using 10 kDa Amicon^®^ Ultra filters (Millipore, Merk Ltda.). The obtained samples were analyzed by UV-Vis spectrophotometry using a microplate reader Synergy^TM^ H1 (BioTek Instrument Inc.). The absorption spectra were registered between 300 and 700 nm, and the emission spectra between 440 and 700 nm after excitation at 400 nm.

### Structural and Chemical Characterization of QDs

The size of biosynthesized QDs was determined by dynamic light scattering (DLS). Purified and concentrated QDs were sonicated for 2 min and then measured in triplicate using a Zetasizer Nano (ZS) (Malvern Instrument Ltd.). High resolution scanning transmission electron microscopy (HR-STEM) was used to confirm nanometric size and chemical composition (FEI Tecnai G2 F20 S-Twin microscope, operated at 200 kV). For these purposes, 2 μL of the purified and concentrated QDs solution was added to a HC300-Cu grid and left to dry. TEM images were processed and analyzed with Digital Micrograph 3.9.0 (Gatan Inc) and The Gimp 2.4.0 software packages. In addition, samples were chemically characterized by Energy-dispersive X-ray spectroscopy (EDS or EDX).

To determine the organic composition of the external layer of QDs, samples werefreeze-dried for 48 h and the powder obtained was mixed with KBr to form a thin pellet. FTIR spectroscopy in a range between 600 and 4000 cm^–1^ was performed using a Nicolet^TM^ iS^TM^10 (Thermo Fisher Scientific Inc.).

### QDs Photo-Stability

Quantum dots samples were normalized by dilution in Milli-Q ultrapure water to 0.06 arbitrary units (A.U.) according to the maximum absorbance peaks (410 and 425 nm for CdS and CdS/CdSe QDs, respectively). QDs were then exposed to constant light (70 mW/cm^2^) and the decay of the peaks was measured every 15 min during 1 h.

### Preparation of the Electrodes and Characterization of the QD Sensitized Solar Cells (QDSSCs)

Quantum dots sensitized solar cells were built following the protocols described by Bang and Kamat (2009) and Órdenes-Aenishanslins et al. (2014, 2016). To prepare the electrodes, fluorine doped tin oxide (FTO) glasses of 20 mm × 20 mm × 2 mm, 13 [Ω/sq] and 85% transmittance were used. A TiO_2_ film prepared with a suspension of nanoparticles (nanopowder, ∼21 nm particle size and anatase crystal structure, Sigma-Aldrich Co.) was applied on the glass by spin coating. The electrode was then sintered at 450°C for 30 min. CdS or CdS/CdSe incorporation to the electrode was obtained by direct adsorption of 10 μL of a 100 mg/mL QDs suspension. The active area of the cells was 1 cm^2^. The counter electrode was prepared using a 50 mM H_2_PtCl_6_⋅6H_2_O solution prepared in isopropanol that was dispersed in an FTO glass by spin coating. Subsequently, this electrode was heated at 400°C for 20 min. Both electrodes were assembled using an inert spacer. Before sealing the cell, a sulfide/polysulfide solution prepared from 1.0 M Na_2_S, 0.1 M S, and 0.1 M NaOH in ultrapure Milli-Q water was added. Characterization of the solar cells was performed under standard conditions of temperature and irradiance using a solar simulator (A1 Solar LightLine, Sciencetech Inc.) as a source of light (100 mW/cm^2^ and AM1.5). A Current-Voltage Measurement System (IV Tester, SSIVT-20C, Sciencetech Inc.) was used to register the current-voltage curves and to obtain the photovoltaic parameters: efficiency (η), fill factor (FF), short-circuit current (Isc), and open circuit voltage (Voc).

## Results

### Biosynthesis of Core/Shell CdS/CdSe QDs

The biosynthesis of CdS/CdSe QDs was divided in 2 steps: First, the synthesis of the CdS core and then the production of the CdSe shell. To determine the best sulfur source for *E. coli* biosynthesis of the CdS core, bacterial cells were treated with different sulfur sources (Na_2_S, L-methionine or L-cysteine) and the production of QDs-mediated fluorescence was evaluated (Supplementary Figure S1). No fluorescence was found at any incubation time when Na_2_S was used. On the other hand, an intense fluorescence emission was obtained when L-cysteine was used as a sulfur source for biosynthesis. In water, QDs production was observed at longer incubation times, between 6 and 24 h. As expected, best results were obtained when Borax-citrate was used as buffer (Monrás et al., 2014). CdS QDs biosynthesized this way are obtained faster and display the characteristic emission colors and intensities of this type of nanoparticles.

**FIGURE 2:**
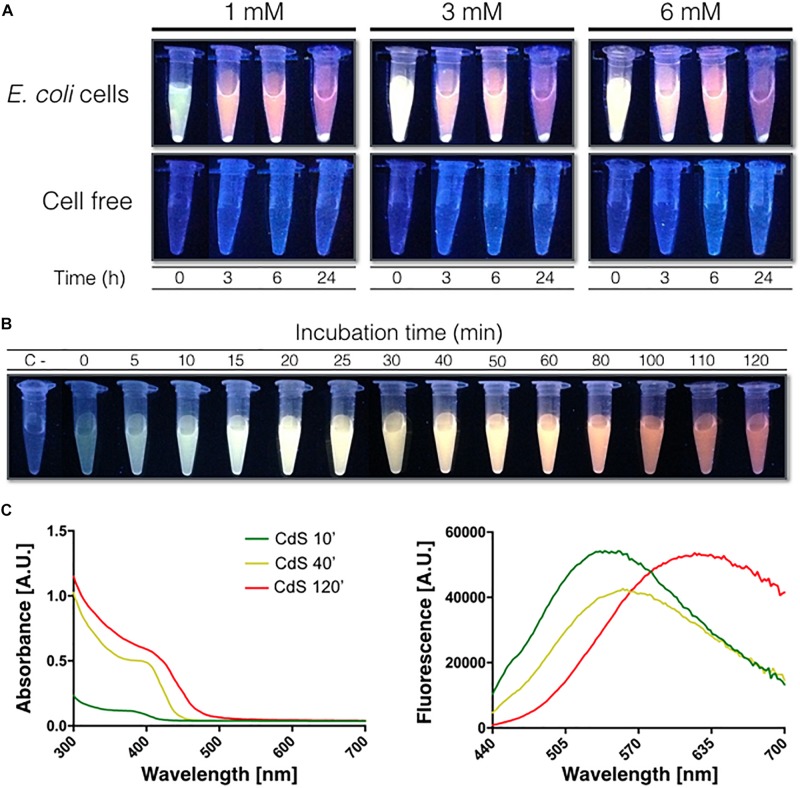
**(A)** Biosynthesis of CdS QDs using Borax-citrate (pH 9.35) as buffer and L-cysteine (1, 3, or 6 mM) as sulfur source. **(B)** Kinetic of the extracellular biosynthesis of CdS QDs in Borax-citrate, 1 mM L-cysteine and 100 μM CdCl_2_. The negative control (C–) corresponds to the reaction in absence of living cells. **(C)** Absorption and fluorescence emission spectra of CdS QD core at 10, 40, and 120 min biosynthesis.

The optimal conditions defined for extracellular CdS core QDs biosynthesis were Borax-citrate as buffer, 1 mM L-cys and 100 μM CdCl_2_ (Figure 2A). Since QDs change their spectroscopic properties once they change their size, and the size increases with time, a kinetic of biosynthesis was performed. When QDs are excited with UV-light, small NPs emit green or blue light, while bigger QDs emit orange, or red light (Alivisatos, 1996). The biosynthesis reaction was performed according to the optimal conditions defined and the extracellular biosynthesis of CdS core QDs was evaluated in culture supernatants exposed to UV (Figure 2B). As expected, green fluorescence was observed at initial time (0–10 min) and then changed to red fluorescence after 120 min reaction.

To further characterize the QDs CdS core, extracellular NPs biosynthesized at 3 time points (10, 40, and 120 min) were purified and analyzed by UV-visible spectrophotometry (Figure 2C). Absorption spectra of biosynthesized QDs at the 3 time points evaluated show the specific signals expected for CdS NPs, with a high absorption in the UV range (between 300 and 400 nm). Different emission peaks were observed for the three QDs, at 540, 560, and 630 nm, respectively. This displacement agrees with the spectral change observed in Figure 2B. The production yield of QDs biosynthesized by our method was determined by calculating the mass of nanoparticles produced in a determined reaction volume. A yield of approximately 0.4–0.6 mg/mL were determined, a result that is in agreement with yields previously obtained by us and others when producing other Cd-nanomaterials using similar protocols (Bao et al., 2010). When the production of QDs was evaluated considering the mg of dry cells used in the biosynthesis, a production yield of 1.54 mg of CdS QDs per mg of dry cells was obtained.

Once the method to biosynthesize the CdS core of QDs was optimized, efforts were directed to incorporate the CdSe shell. Again, 3 representative times were selected (20, 40, and 60 min) and at these times 3 different concentrations of Na_2_SeO_3_ were tested (1, 10, and 100 μM) (Supplementary Figure S2A). As has been reported before for CdS/CdSe Core/Shell chemical synthesis, the formation of a shell can be evidenced by changes in the spectroscopic properties of the nanocrystal, particularly a red shift in absorption and emission (Battaglia et al., 2003; Xu et al., 2005; Du et al., 2010). A red shift in fluorescence emission was observed when Na_2_SeO_3_ 1 or 10 μM was added to biosynthesized green, yellow or orange CdS QDs (core) produced after 20, 40, or 60 min of reaction, respectively (Supplementary Figure S2). No fluorescence was observed when Na_2_SeO_3_ 100 μM was incorporated to the reaction.

**FIGURE 3:**
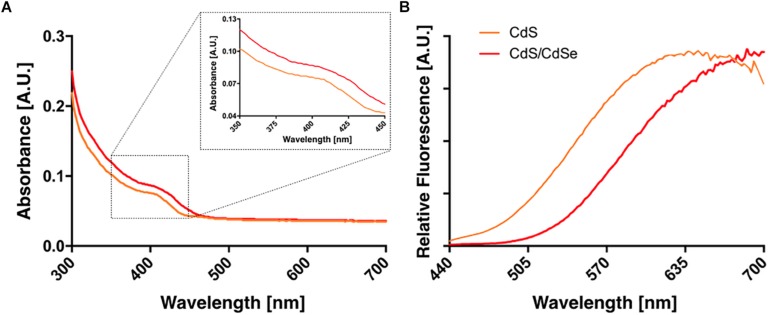
Absorption **(A)** and fluorescence emission spectra **(B)** of biologically produced CdS and CdS/CdSe QDs. An inset is shown in left to emphasize the spectral shift.

The absorption and emission spectra of QDs produced in presence of Se shows a displacement to superior wavelengths when compared to conditions when no Se was incorporated to the reaction (Figure 3). This red-displacement in absorption and emission spectra is characteristic of Core/Shell QDs (Battaglia et al., 2003; Xu et al., 2005; Du et al., 2010), and strongly suggest the generation of Core/Shell CdS/CdSe QDs by bacterial cells.

To confirm the importance of living bacterial cells in the synthesis of CdS/CdSe QDs, the same experiments were performed but in presence of heat-inactivated cells or cell extracts. Even when the formation of the CdS core could be seen when using cell extracts, none of the biosynthethic conditions allowed the generation of the Core/Shell structure, determining a strict requirement of the presence of living cells to synthesize the Core/Shell CdS/CdSe QDs (not shown). A production yield of 3.22 mg of CdS/CdSe QDs/mg of dry cells was obtained.

### Characterization of Core/Shell Cds/CdSe QDs Biosynthesized by *E. coli*

**FIGURE 4:**
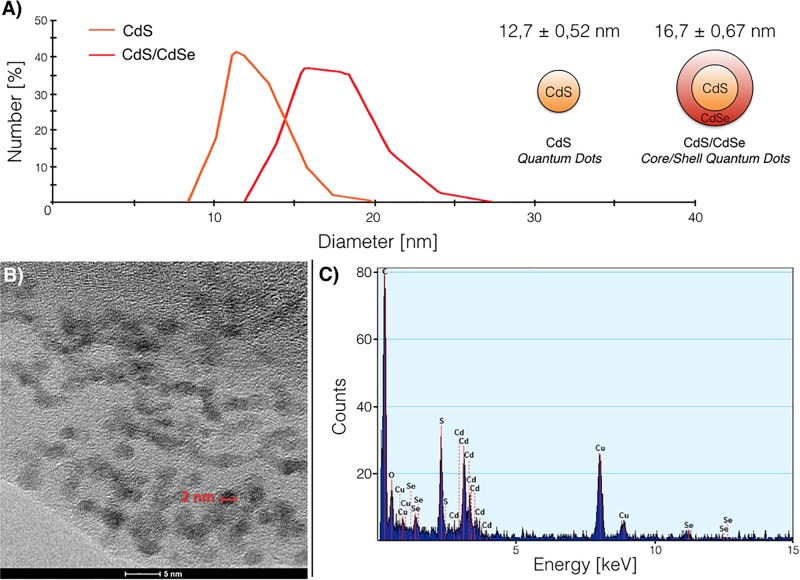
Characterization of biosynthesized quantum dots (QDs). **(A)** Size distribution of CdS and CdS/CdSe biosynthesized QDs determined by DLS. **(B)** TEM images showing the size of individual CdS/CdSe nanoparticles. **(C)** EDS composition analysis of CdS/CdSe QDs.

The size of biosynthesized QDs was analyzed by DLS. Average hydrodynamic sizes of 12.7 and 16.7 nm were determined for CdS and CdS/CdSe QDs (Figure 4A), which is in agreement with the size previously determined for CdS QDs biosynthesized by different microorganisms (Kang et al., 2008; Gallardo et al., 2014; Ulloa et al., 2016, 2018). As expected, Core/Shell QDs are bigger than QDs composed only by the CdS core. The nanometric size and shape of the CdS/CdSe QDs was corroborated by transmission electron microscopy (TEM) (Figure 4B). Core/Shell QDs are mainly grouped in clusters, present a spherical morphology and average size below 5 nm. The chemical composition of biosynthesized Core/Shell QDS was determined by EDS analysis revealing the presence of Cd, S, and Se (Figure 4C) in 40.7, 55.5, and 3.85 wt%, respectively, which are in agreement with Cd:S:Se ratios previously reported for CdS/CdSe QDs (Coria-monroy et al., 2015).

**FIGURE 5:**
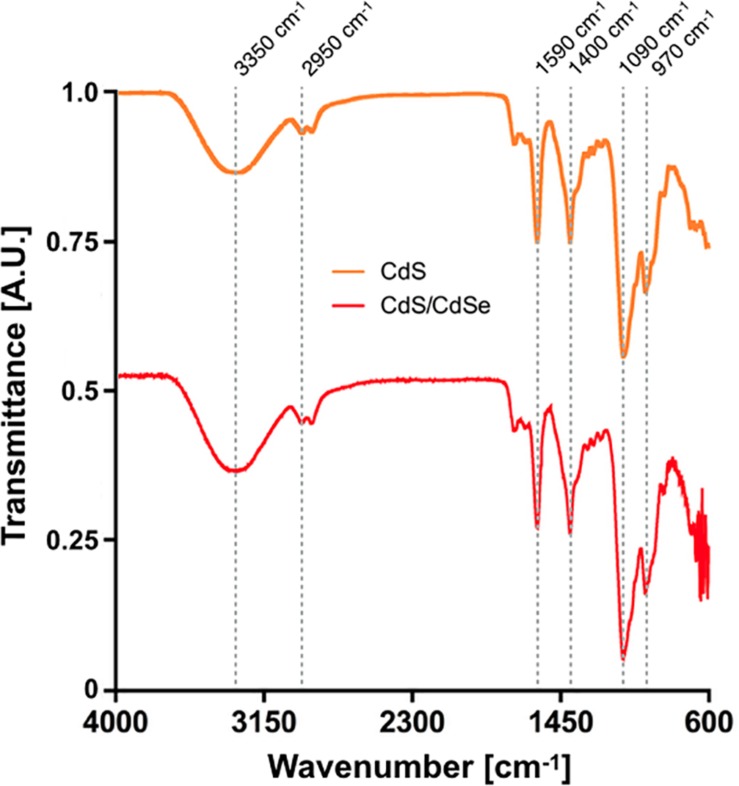
FTIR spectra of biosynthesized CdS and CdS/CdSe QDs.

One of the characteristics of biosynthesized QDs is the presence of biomolecules covering the nanostructure that in some cases determine their properties (Plaza et al., 2016; Ulloa et al., 2016; Bruna et al., 2019). The organic composition of CdS and CdS/CdSe QDs biosynthesized by our method was analyzed by FTIR spectroscopy (Figure 5). Interestingly, the spectra of both QDs is almost identical, indicating that the same biomolecules compose the nanostructure despite the presence of an external shell. The broad peak in 3350 cm^–1^ represents hydroxyl groups in the structure. Bands around 2950 cm^–1^ correspond to C-H interactions, like CH_2_ and CH_3_ in aliphatic hydrocarbons. A band at 1090 cm^–1^ corresponds to C-N vibrations possibly of aliphatic amines. Near 1590 and 1400 cm^–1^ there are signals indicating vibrations of C = O and N-H from amines and amides. Finally, peaks at 1590, 1400, and 970 cm^–1^ could also indicate the presence of C = C double bonds, either from a ring or aliphatic chain. All these signals can be attributed to the presence of biomolecules bound to CdS and CdS/CdSe QDs. These biomolecules probably correspond to peptides or proteins that could participate in the nucleation process and/or stabilizing the nanoparticles. It is expected that enzymes located on the cell membrane or excreted to the extracellular media could participate in nanoparticle synthesis (Raouf Hosseini and Nasiri Sarvi, 2015).

**FIGURE 6:**
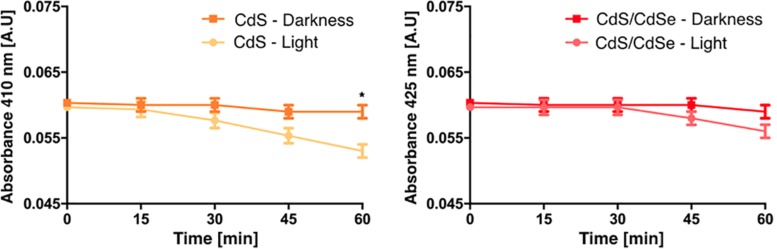
Photo-stability of biosynthesized CdS QD core or CdS/CdSe QDs. The decay of absorbance peaks was determined under light (70 mW/cm^2^) and darkness conditions (^*^p < 0.0001).

The presence of a shell covering the CdS core could add a physical barrier between the optically active QD and the surrounding media, and this could improve the resistance to photobleaching and also enlarge their lifespan (Reiss et al., 2009; Vasudevan et al., 2015). To determine if the CdS/CdSe QDs are more resistant to photobleaching, biosynthesized QDs were exposed to constant light (70 mW/cm^2^) and the decay of the maximum absorbance peaks was measured every 15 min for 1 h (Figure 6). The decay of CdS QDs is more pronounced than the decay of CdS/CdSe QDs, with a significant difference in photo-stability after 60 min. This characteristic is an important advantage when considering the application of biological QDs in QDSSCs.

### Biological Core/Shell QDs as Sensitizers in Solar Cells

**FIGURE 7:**
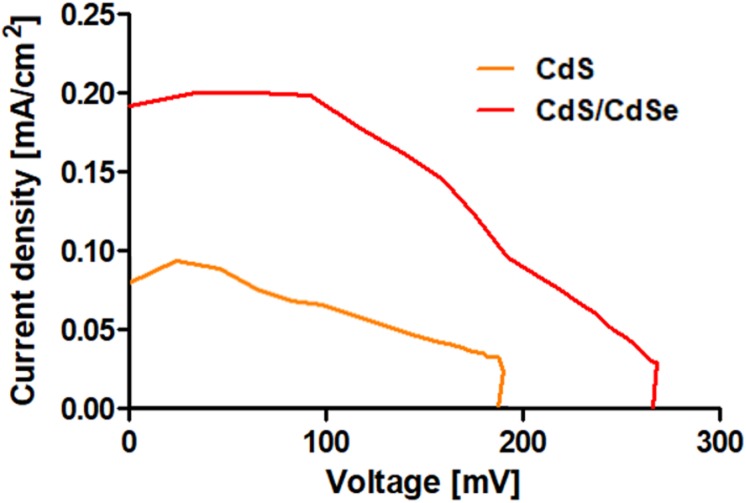
Current- voltage curves for solar cells produced using biosynthesized CdS or CdS/CdSe QDs as photosensitizers.

Due to the promising properties observed in the CdS/CdSe Core/Shell QDs produced, these biological QDs were tested for the first time in a QDSSC. Solar cells were built following the protocols described by Bang and Kamat (2009) and Órdenes-Aenishanslins et al. (2014, 2016), and the characterization was performed using a solar simulator as light source. To compare the efficiencies of conversion, solar cells were constructed using biosynthesized CdS QDs or CdS/CdSe Core/Shell as photosensitizers. The current-voltage curves of the constructed cells (Figure 7) show that the photovoltaic devices present the characteristic shape expected for QDSSCs (Sarker et al., 2015) and that the use of Core/Shell QDs as photosensitizers improve the properties of the cell when compared to biological CdS QDs but also when compared to chemical CdTe QDs.

Photovoltaic parameters such as short-circuit current (Isc) and open circuit voltage (Voc) were determined for both cells (Table 1). The CdS QDs-sensitized solar cell presented a Voc of 279 mV, an Isc of 8.19 × 10^–2^ mA/cm^2^ and an efficiency of 8.01 × 10^–3^%. On the other side, the CdS/CdSe solar cell showed a Voc of 209 mV, an Isc of 2.03 × 10^–1^ mA/cm^2^, and an efficiency of 2.0 × 10^–2^%. A 2.5 times increase in efficiency was determined between biological Core/Shell QDs and CdS QDs. In addition, when compared with solar cells sensitized with chemical CdTe QDs (regularly used as sensitizers in solar cells), better photovoltaic parameters were determined in presence of biological core shell QDs (efficiency increased 5.36 times). Interestingly, similar values were observed when biological CdS QDs and chemical CdTe QDs were used, validating the use of biologically produced nanomaterials as photosensitizers in QDSSCs.

**TABLE 1:** Photovoltaic parameters obtained from the current-voltage curves for solar cells produced using biosynthesized CdS or CdS/CdSe QDs as photosensitizers.

**Sensitizer**	**Short current density *J*_sc_ [mA/cm^2^]**	**Open circuit voltage *V*_oc_ [mV]**	**Fill factor [%]**	**Maximum power *P*_max_ [W]**	**Efficiency *η* [%]**
CdTe	3.66 × 10^–2^ ± 5.14 × 10^–4^	184 ± 03	55.4 ± 0.78	3.73 × 10^–6^ ± 6.30 × 10^–8^	3.73 × 10^–3^ ± 6.30 × 10^–5^
CdS	8.19 × 10^–2^ ± 3.46 × 10^–3^	279 ± 26	42.8 ± 4.10	8.08 × 10^–6^ ± 7.40 × 10^–7^	8.01 × 10^–3^ ± 7.40 × 10^–4^
CdS/CdSe	2.03 × 10^–1^ ± 9.73 × 10^–3^	209 ± 11	42.2 ± 14.7	2.00 × 10^–5^ ± 1.90 × 10^–6^	2.00 × 10^–2^ ± 1.90 × 10^–3^

*A chemical sensitizer was also evaluated as control, CdTe.*

This is the first work reporting the construction of QDSSC using biosynthesized Core/Shell CdS/CdSe QDs as sensitizers and, as expected, they presented a better performance in terms of efficiency than solar cells sensitized with CdS QDs.

## Discussion

The aim of this work was to develop a biological method that allows the production of Core/Shell CdS/CdSe QDs specially designed to be used as photosensitizers in solar cells. To achieve this, the influence of different buffers and sulfur sources was explored to determine the best conditions to produce high quantities of CdS QDs with characteristics that favor the incorporation of a shell. The optimal condition determined for the *E. coli* production of CdS QDs involved the use of Borax citrate as buffer and L-cysteine as a sulfur source. Borax citrate buffer also has been described as an optimal buffer for chemical and biological synthesis of other metallic nanoparticles (Monras et al., 2012).

Recently, the relevance of hydrogen sulfide (H_2_S) in the extracellular biosynthesis of CdS QDs by Antarctic bacteria (Gallardo et al., 2014; Plaza et al., 2016) and bacteria isolated from the Atacama desert (Bruna et al., 2019) has been described. A basic pH of the buffer (9.35) favors the deprotonation of H_2_S to generate S^2–^ in solution thus favoring the interaction with Cd^2+^ to form nanoparticles. In accordance to this, we determined that none of the buffers that tamponade at pHs below 8.00 gave better results regarding CdS/CdSe QDs biosynthesis than Borax citrate (Supplementary Figure S1).

Our biological method to synthesize CdS and CdS/CdSe QDs requires the presence of active bacterial cells since QDs biosynthesis was not observed when heat inactivated cells or cells extracts were used. In cysteine-mediated QDs biosynthesis reactions, the participation of enzymes like cysteine desulfhydrase are needed to produce H_2_S (through L-cysteine) that will contribute with sulfur production (Bai et al., 2009; Iravani, 2014). Also, a cover of peptides or proteins provided by the cell are thought to stabilize the nanostructure (Durán et al., 2011; Monras et al., 2012; Órdenes-Aenishanslins et al., 2014; Raouf Hosseini and Nasiri Sarvi, 2015). Besides, the presence of living bacteria could be mediating the reduction of selenite to selenide due to reductases of the selenium metabolism (Turner et al., 1998). However, the proteins involved in extracellular biosynthesis of Cd-based QDs are still unknown.

Synthesis of QDs was mainly observed in the supernatant (extracellular), which is an advantage in terms of purification, and also for the photo-physical and structural characterization. A yield of 1.54 mg CdS QDs per mg of dry cells was obtained by the described method. Interestingly, the production yield was duplicated with the protocol to biosynthesize core shell CdS/CdSe QDs (3.22 mg of QDs/mg of dry cells). This result reinforces the advantages and potential for industrial applications of our method to biosynthesize core shell QDs since in addition to the improved properties and performance as sensitizer, the procedure increases the production yield.

The DLS analysis indicated that CdS QDs have an average size of 12.7 nm, while de CdS/CdSe QDs an average of 16.7 nm. The high polydispersity observed in the samples is expected for biosynthesized QDs, suggesting that nanoparticles are produced at different times after exposure of cells to the metal (Sweeney et al., 2004; Park et al., 2010; Monras et al., 2012; Yan et al., 2014). This agrees with the presence of small nanoparticles (2 nm) and also grouped nanoparticles observed in the TEM images. According to FTIR spectra, these nanoparticles are covered by organic matter, obtaining signals that can be attributed to biomolecules like peptides or proteins. These biomolecules could be acting supporting the nucleation of QDs or, as mentioned before, as stabilizers of the nanostructure (Durán et al., 2011; Monras et al., 2012; Órdenes-Aenishanslins et al., 2014).

In this study, we developed a tunable biosynthesis method that allows a tight regulation of QDs properties such as size, fluorescence emission, and composition. In this aspect, other QDs with tunable characteristics have been produced using extremophile microorganisms that tolerate low temperatures (Gallardo et al., 2014), acidic pH (Ulloa et al., 2016, 2018), and high salt concentrations (Bruna et al., 2019). Understanding the molecules involved in biosynthesis reactions, would allow a better control of the properties of QDs thus favoring their application in different technologies.

Various studies have pointed the advantages in photo resistance of Core/Shell QDs when compared to regular QDs, a condition associated with the presence of an extra semiconductor material (Peng et al., 1997, 2007; Aldeek et al., 2011; Doskaliuk et al., 2016). In this context, we hypothesized that the presence of an additional organic layer, characteristic of biosynthesized nanomaterials, could also contribute to improve the photo-stability of Core/Shell QDs. Our results indicated that biologically produced Core/Shell QDs are more photo-stable than CdS QDs, which is a key characteristic for their technological application in solar cells and in bioimaging.

Based on this, biologically produced CdS/CdSe QDs were evaluated as photosensitizers in QDSSCs. A significant improvement in efficiency was observed in solar cells photosensitized with Core/Shell QDs when compared with biosynthesized CdS QDs (2.5 times improvement). Interestingly, the same behavior was observed when compared with CdTe QDs produced by a chemical method. This improvement is explained fundamentally because the shell act as a layer that tends to catch, retain and order the electrons on the superior layers, helping to direct the charges to the electrode, and avoiding alternative decays (Reiss et al., 2009; Vasudevan et al., 2015).

Altogether, obtained results validate the application of biosynthesized Core/Shell QDs in solar cells, but also stablish our biosynthesis method as a valid alternative for the production of different nanomaterials with improved properties. In addition, the described protocol allows a tight control of the properties of biosynthesized NPs favoring the generation of biological nanoparticles for specific applications.

This is the first time that biosynthesized CdS/CdSe Core/Shell QDs are produced in biological systems and tested in QDSSCs. Presented results validate biological core shell QDs as a promising alternative for QDSSCs in terms of photovoltaic parameters and environmental impact. The results presented here will contribute to the development of new biological methods to synthesize QDs with properties that will favor their application in QDSSCs and other technological applications.

## Data Availability

The raw data supporting the conclusions of this manuscript will be made available by the authors, without undue reservation, to any qualified researcher.

## Author Contributions

NÓ-A, DB, and JP-D conceived and designed the study. NÓ-A and GA-O conducted the microbiology experiments and analyzed the data. RE-G developed the TEM studies. CQ, GA-O, and JP-D wrote the manuscript. All authors contributed to the scientific discussion and revision of the manuscript.

## Conflict of Interest Statement

The authors declare that the research was conducted in the absence of any commercial or financial relationships that could be construed as a potential conflict of interest.

## Supplementary Material

FIGURE S1 Extracellular biosynthesis of CdS QDs using different concentrations of sodium sulfur, L-methionine, and L-cysteine as sulfur source (the oxidation state of the sulfur is indicated). The effect of water and three buffers on the biosynthesis were also tested. As a negative control, the synthesis was tested in the absence of bacterial cells.

FIGURE S2 **(A)** Effect of time and Na_2_SeO_3_ concentration on CdS/CdSe core/shell QDs biosynthesis. *E. coli* cultures biosynthesizing CdS QDs (20, 40, or 60 min biosynthesis, left side) were exposed to 1, 10, or 100 μM Na_2_SeO_3_. Absorption spectra of CdS/CdSe QDs after adding 1, 10, or 100 μM Na_2_SeO_3_ to the CdS QD core for **(B)** 20, **(C)** 40, or **(D)** 60 min of incubation. An inset is shown to emphasize the spectral shift.
