# Supplementary figures and images for: Biological Synthesis of CdS/CdSe Core/Shell Nanoparticles and Its Application in Quantum Dot Sensitized Solar Cells
Source: Front Microbiol. 2019 Jul 11;10:1587. doi: 10.3389/fmicb.2019.01587 (PMC6637821; doi:10.3389/fmicb.2019.01587)

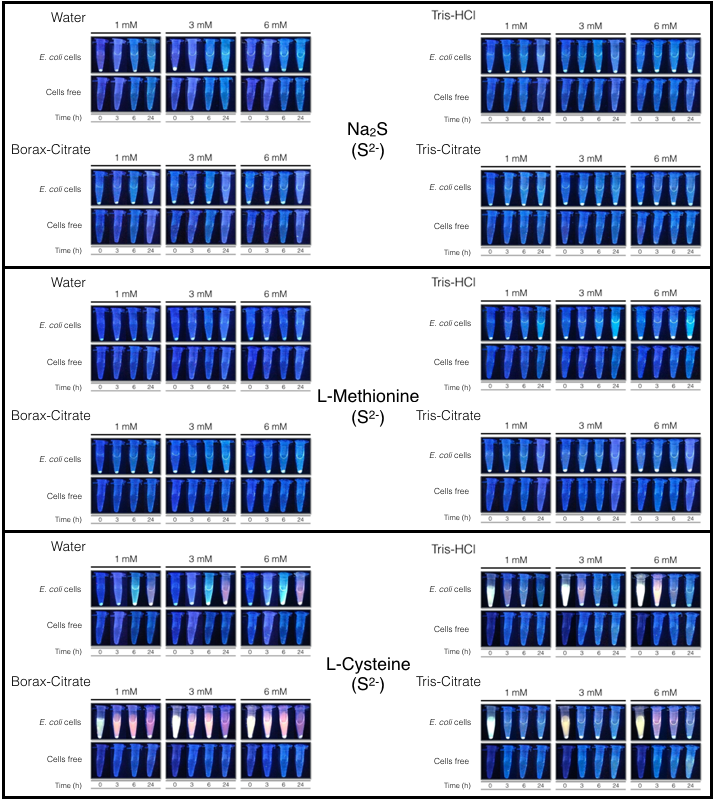

Supplement: FIGURE S1 — Extracellular biosynthesis of CdS QDs using different concentrations of sodium sulfur, L-methionine, and L-cysteine as sulfur source (the oxidation state of the sulfur is indicated). The effect of water and three buffers on the biosynthesis were also tested. As a negative control, the synthesis was tested in the absence of bacterial cells. [file Image_1.TIFF]

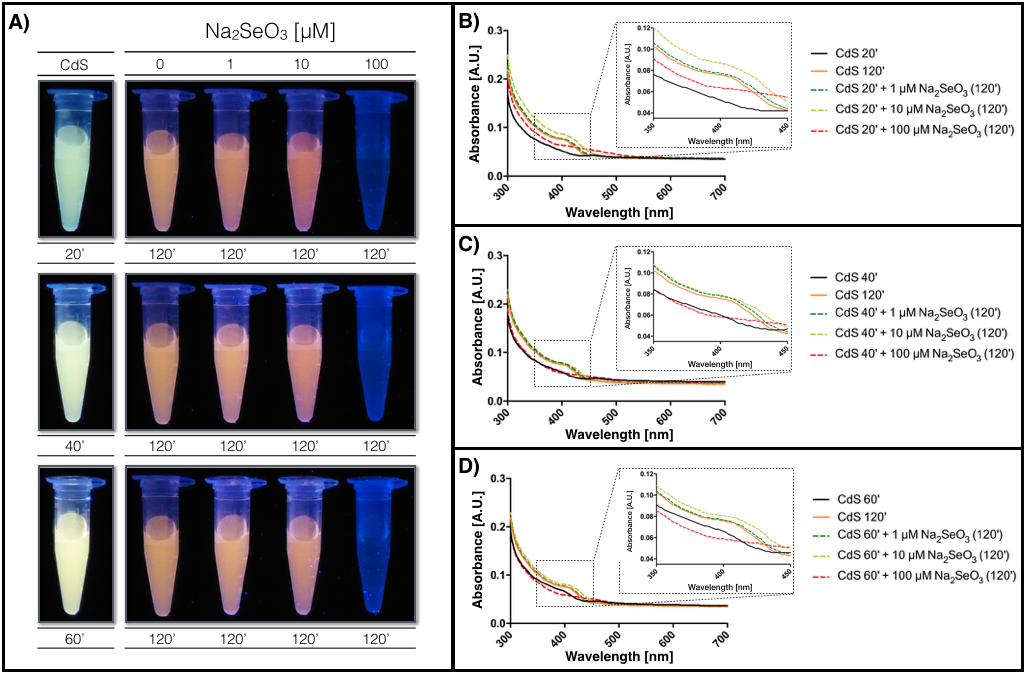

Supplement: FIGURE S2 — (A) Effect of time and Na2SeO3 concentration on CdS/CdSe core/shell QDs biosynthesis. E. coli cultures biosynthesizing CdS QDs (20, 40, or 60 min biosynthesis, left side) were exposed to 1, 10, or 100 μM Na2SeO3. Absorption spectra of CdS/CdSe QDs after adding 1, 10, or 100 μM Na2SeO3 to the CdS QD core for (B) 20, (C) 40, or (D) 60 min of incubation. An inset is shown to emphasize the spectral shift. [file Image_2.TIFF]
